# KS23, a novel peptide derived from adiponectin, inhibits retinal inflammation and downregulates the proportions of Th1 and Th17 cells during experimental autoimmune uveitis

**DOI:** 10.1186/s12974-019-1686-y

**Published:** 2019-12-28

**Authors:** Tian Niu, Lu Cheng, Hanying Wang, Shaopin Zhu, Xiaolu Yang, Kun Liu, Huiyi Jin, Xun Xu

**Affiliations:** 1 Department of Ophthalmology, Shanghai General Hospital, Shanghai Jiao Tong University School of Medicine, Shanghai, China; 2 Shanghai Key Laboratory of Ocular Fundus Diseases, Shanghai, China; 3 Shanghai Engineering Center for Visual Science and Photomedicine, Shanghai, China

**Keywords:** Experimental autoimmune uveitis, KS23, Adiponectin, AMPK, Th17 cells, Peptide, Th1 cells

## Abstract

**Background:**

Uveitis is a potentially sight-threatening form of ocular inflammation that affects the uvea in the wall of the eye. Currently available treatments for uveitis have exhibited profound adverse side effects. However, KS23 is a novel 23-amino-acid anti-inflammatory peptide derived from adiponectin that may have the capability to function as a safe alternative to these existing treatment options. We, therefore, evaluated the preventive effect of KS23 in experimental autoimmune uveitis (EAU).

**Methods:**

EAU was induced in mice via immunization with the peptide interphotoreceptor retinoid binding protein 161–180 (IRBP161–180). KS23 was then administered every 2 days via intraperitoneal injection to induce protection against EAU. Clinical and histopathological scores were employed to evaluate the disease progression. Inflammatory cytokines were also quantified using ELISA, and the expression levels of specific chemokines and chemokine receptors were assessed via qRT-PCR. In addition, the proportions of Th1 and Th17 cells were detected via flow cytometry, and the expression levels of specific proteins were quantified from the retina of mice using western blot analysis, to elucidate the specific mechanism of action employed by KS23 to suppress the inflammation associated with EAU.

**Results:**

KS23 was found to significantly improve EAU-associated histopathological scores, while decreasing the expression of pro-inflammatory cytokines (IFN-γ, TNF-α, IL-6, and IL-17A), chemokines (LARC, RANTES, MIG, IP-10), and chemokine receptors (CCR6 and CXCR3). The proportions of Th1 and Th17 cells were also suppressed following intraperitoneal injection with KS23. The anti-inflammatory mechanism employed by KS23 was determined to be associated with the activation of AMPK and subsequent inhibition of NF-κB.

**Conclusions:**

KS23 decreased the proportions of Th1 and Th17 cells to effectively ameliorate the progression of EAU. It may, therefore, serve as a promising potential therapeutic agent for uveitis.

## Background

Uveitis, comprising a diverse group of intraocular inflammatory diseases, is one of the leading causes of reversible and long-term visual impairment [1, 2]. Uveitis is classified based on the etiology of inflammation, as either infectious or non-infectious. The latter is induced by systemic immune-mediated or systemic autoimmune factors [3]. The Standardization of Uveitis Nomenclature Working Group Guidelines has recommended the use of corticosteroids as the first-line therapy for patients in the active phase of uveitis [4]. However, prolonged treatment with corticosteroids is associated with several ocular and systemic adverse side effects and possible relapse as the corticosteroids are withdrawn [5, 6]. The emergence of new immunomodulatory and immunosuppressive therapies has proven to be more effective while exhibiting less toxicity [7]. Thus, agents capable of regulating specific molecular and cellular targets may provide more suitable treatment options for uveitis, as compared to the more broadly acting corticosteroids.

T helper (Th) cells are differentiated from naive CD4^+^ T cells and regulate inflammatory responses as well as general immunity. Although the pathogenesis of human autoimmune uveitis is not fully understood, the association between the development of uveitis and Th cell responses has been established as a vital contributor to the etiology of autoimmune uveitis [8, 9]. Moreover, the differentiation and activation of pro-inflammatory destructive Th cell subsets, including Th type 1 (Th1) and Th type 17 (Th17), have been shown to induce secretion of pro-inflammatory cytokines that are capable of breaching the blood-retinal barrier (BRB) [10]. These pro-inflammatory cytokines recruit macrophages, resulting in intraocular inflammation [11]. Although the pathology of experimental autoimmune uveitis (EAU) cannot exactly represent human uveitis due to the structural difference between human eyes and those of mice, these intricate mechanisms have been partly characterized through the use of EAU, which closely resembles human uveitic diseases of an autoimmune nature, particularly in the patients’ immune responses to retinal antigens [12, 13].

Adiponectin (APN) is an adipokine hormone produced by several tissues that circulates throughout the blood plasma [14]. APN has a broad range of functions including insulin sensitization, metabolic regulation, and anti-inflammatory effects [15]. The role of APN as a prominent inhibitor of immune responses has been investigated in various autoimmune disease models, such as multiple sclerosis and psoriasiform dermatitis [16, 17]. The globular C-terminal domain of APN (gAPN) is one of the key APN isoforms responsible for eliciting various biological [18]. Further, in an in vivo APN-deficient vascular inflammation model, treatment with gAPN was found to significantly reduce leukocyte-endothelium interactions and adhesiveness [19]. Moreover, the CD4^+^ effector cells, which are considered central in both human uveitis and EAU, were also suppressed by gAPN in an experimental model of autoimmune encephalomyelitis [20, 21].

In this study, we assessed the efficacy of a novel 23-amino-acid peptide (KS23) derived from gAPN in the amelioration of symptoms associated with experimental autoimmune uveitis (EAU). EAU was induced with peptide interphotoreceptor retinoid binding protein 161–180 (IRBP161–180, R161). We also sought to elucidate the mechanism employed by KS23 in the prevention of EAU by examining changes in the expression of proteins associated with the AMP-activated protein kinase (AMPK) and nuclear factor-kappa B (NF-κB) signaling pathways.

## Methods

### Animals

B10RIII mice (8-week-old) were procured from the Jackson Laboratory (Bar Harbor, ME, USA). All the mice were maintained in a 12-h light/12-h dark cycle under specific pathogen-free conditions. All procedures involving animals were in accordance with the *ARVO Statement for the Use of Animals in Ophthalmic and Vision Research*.

### Peptide synthesis

According to the characteristics of the APN globular C-terminal domain, our study focused on one conserved amino acid sequence (KS23: KDKAMLFTYDQYQENNVDQASGS). The scrambled peptide, TQ23 (TADYNGMKANVDQQYSESKDFLQ), was used as a control. The peptide administered to induce EAU consisted of the 161–180 amino acid sequence of the IRBP. The specific sequence used was as follows: SGIPYIISYLHPGNTILHVD. The peptides were synthesized and purified using an automatic peptide synthesizer (Symphony; Protein Technologies, Tucson, AZ), which is a high-efficiency, solid-phase method.

### EAU induction and evaluation

To induce EAU, B10RIII mice were injected subcutaneously with 100 μL of 20 μg R161 peptide, suspended in phosphate-buffered saline (PBS) and emulsified in Freund’s complete adjuvant (CFA, 1:1 v/v, BD Pharmingen, San Diego, CA, USA), containing *Mycobacterium tuberculosis* strain H37RA (2.5 mg/mL, BD Pharmingen). The mice were scored by fundoscopy and histologic examination in accordance with the previously described protocols [12].

### Experimental design

To evaluate the effect and most efficacious concentration of KS23, mice were first induced with EAU and subsequently intraperitoneally injected with KS23 (5 mg/kg, 10 mg/kg, or 15 mg/kg), every 2 days. The clinical signs were scored following each immunization. The eyeballs were collected on day 28, and histological scores were determined via hematoxylin and eosin (H&E) staining. Secondly, we randomly assigned the mice into four groups, namely the control group (no EAU), EAU group, EAU + KS23 group, and EAU + TQ23 group. The mice in the EAU + KS23 group were intraperitoneally injected with KS23 at the optimum dosage, according to results obtained from the initial histological analysis. The mice in the EAU + TQ23 group received TQ23 intraperitoneal injections with the same dosage that was administered to the KS23 group. The control and EAU groups also received intraperitoneal injections with equal volumes of PBS. The mice in the control group received identical prevention as the EAU group saved for the administration of R161. Clinical and histological scores, as well as additional analyses, were all performed on samples collected at the peak of EAU manifestation, as determined during the initial histological analysis.

### Enzyme-linked immunosorbent assays

Iris ciliary body (ICB)-retina complexes were isolated from the eyeballs and placed in 100 μL lysis buffer (Merck, Darmstadt, Germany) containing a Protease Inhibitor Cocktail (Roche, Mannheim, Germany). The lysate was then sonicated and centrifuged at 12,000 rpm for 10 min at 4 °C to collect the supernatants. The protein concentrations were assessed using a bicinchoninic acid kit (Sigma-Aldrich). ELISA kits (R&D Systems, Minneapolis, MN, USA) were used to evaluate the expression levels of interferon gamma (IFN-γ), tumor necrosis factor alpha (TNF-α), interleukin 6 (IL-6), and interleukin 17A (IL-17A) in the ICB and retina complex, according to the manufacturer’s instructions.

### Quantitative real-time polymerase chain reaction analysis

The retinas were isolated, and the relative mRNA expression levels of liver and activation-regulated chemokine (LARC/CCL20); regulated upon activation, normal T cell expressed, and secreted (RANTES/CCL5); monokine induced by gamma interferon (MIG/CXCL9); IFN-γ-inducible protein 10 (IP-10/CXCL10); C-C motif chemokine receptor 6 (CCR6); and C-X-C motif receptor 3 (CXCR3) were quantified via qRT-PCR. The total RNA was extracted using Trizol reagent (Invitrogen, Carlsbad, CA, USA), and complementary DNA (cDNA) was synthesized using the RT Master Mix (Takara, Dalian, China), as per the manufacturer’s protocol. qRT-PCR was performed using an ABI Prism 7500 Sequence Detection System (Applied Biosystems, Foster City, CA), wherein β-actin was used as the housekeeping gene.

The specific primers used for qRT-PCR were as follows: LARC—(forward) 5′-ACTGTTGCCTCTCGTACATACA-3′ and (reverse) 5′-GAGGAGGTTCACAGCCCTTTT-3′; RANTES—(forward) 5′-GCAAGTGCTCCAATCTTGCA-3′ and (reverse) 5′-CTTCTCTGGGTTGGCACACA-3′; MIG—(forward) 5′-CTTTTCCTTTTGGGCATCATCT-3′ and (reverse) 5′-TCGTGCATTCCTTATCACTAGGG-3′; IP-10—(forward) 5′-GCCGTCATTTTCTGCCTCAT-3′ and (reverse) 5′-GCTTCCCTATGGCCCTCATT-3′; CCR6—(forward) 5′-CCTCACATTCTTAGGACTGGAGC-3′ and (reverse) 5′-GGCAATCAGAGCTCTCGGA-3′; CXCR3—(forward) 5′-TGCTGTGCTACTGAGTCAGCG-3′ and (reverse) 5′-TACAGCCAGGTGGAGCAGG-3′; and β-actin—(forward) 5′-CTAAGGCCAACCGTGAAAG-3′ and (reverse) 5′-ACCAGAGGCATACAGGGACA-3′.

### Flow cytometric analysis

Single-cell suspensions were isolated from the mouse spleens and perform flow cytometric analysis immediately. For Th1 and Th17 cell staining, isolated cells were stimulated by anti-CD3/CD28 dynabeads (Gibco, Grand Island, NY, USA, 11452D) as per instructions for 24 h at 37 °C and 5% CO2 in DMEM (Gibco) supplemented with 10% fetal bovine serum (FBS; ScienCell, San Diego, CA, USA) and antibiotics (100 U/mL penicillin and 100 mg/mL streptomycin). During the last 6 h, Brefeldin A (eBioscience, San Diego, CA, USA) 10 μg/mL was added. Cultured cells were washed twice in PBS and resuspended in stain buffer (BD Pharmingen). The cells were first stained with Fixable Viability Stain (BD Pharmingen, 564406) and then washed twice in PBS. Afterwards, cells were stained for surface antigens CD3 (anti-mouse CD3 PerCP-Cy5.5 antibody, BD Pharmingen, 561108) and CD4 (anti-mouse CD4 FITC antibody, BD Pharmingen, 557307), then cells washed in PBS and permeabilized via BD Perm/Fix kit (BD Biosciences, 562574) according to the manufacturer’s instructions. For Th1 cell staining, cells were incubated with anti-mouse IFN-γ APC (BD Pharmingen, 554413) and anti-mouse T-bet PE (BD Pharmingen, 561268) antibodies. For Th17 cell staining, cells were incubated with anti-mouse IL-17A APC (eBioscience, 17-7177-81) and anti-mouse ROR-γt PE (eBioscience, 12-6988-80) antibodies. For T regulatory cell (Treg) cell staining, cells without stimulation were stained with Fixable Viability Stain as described above, followed by staining for surface antigens including CD3, CD4, and CD25 (anti-mouse CD25 APC antibody, BD Pharmingen, 557192). After permeabilization, cells were incubated with anti-mouse Foxp3 PE (BD Pharmingen, 560408) antibodies. Data acquisition was performed on a BD FACSCalibur (Becton Dickinson, Mount View, CA, USA). Single-stained samples of all fluorochromes were used to establish an appropriate compensation matrix. For negative control, intracellular and extracellular isotype controls (BD Pharmingen, 561108, 553988, 554686, 559320, 550884, 555848; eBioscience, 17-4321-81, 12-4321-80) were used. 1 × 10^5^ events were obtained per sample and analyzed by using FlowJo 10.4 (Tree Star, Ashland, OR) as outlined (Additional file 1: Figure S1). We defined cell compartments as follows: Th1, CD3^+^CD4^+^ IFN-γ^+^T-bet^+^; Th17, CD3^+^CD4^+^ IL-17A^+^ ROR-γt ^+^; and Treg, CD3^+^CD4^+^ CD25^+^Foxp3^+^.

### Western blot analysis

Retinas were harvested, sonicated, and centrifuged, and the proteins were extracted using a bicinchoninic acid kit (Sigma-Aldrich, St. Louis, MO, USA). The protein lysates were loaded and separated via sodium dodecyl sulfate-polyacrylamide gel electrophoresis (SDS-PAGE), followed by electrophoretic transfer to polyvinylidene difluoride (PVDF) membranes (Millipore, Billerica, MA, USA). After blocking with 5% bovine serum albumin (BSA) for 1 h, the membranes were incubated overnight at 4 °C with primary antibodies specific for phospho-AMPK, AMPK, sirtuin 1 (SIRT1), peroxisome proliferator-activated receptor gamma (PPAR-γ), acetyl-NF-κB (Ac-NF-κB) p65, NF-κB p65, β-actin (Cell Signaling Technology; Beverly, MA, USA, 2535, 5831, 9475, 2435, 12629, 8242, 3700) and adiponectin receptor 1 (AdipoR1), and ROR-γt (Abcam; Cambridge, MA, USA, ab126611, ab207082). After washing the membranes at room temperature, secondary anti-rabbit or anti-mouse antibodies (Cell Signaling Technology) were added for 1 h. The membranes were then washed, and the bands were visualized using enhanced chemiluminescence (ECL) western blotting substrate (Millipore, Billerica, MA, USA). Images were analyzed with the ImageJ software (National Institutes of Health; Bethesda, MD). Each experiment was repeated a minimum of three times.

### Statistical analyses

The data were analyzed with Prism 6.0 (GraphPad Software, San Diego, CA, USA) and presented as mean ± standard deviation (SD). The normality was analyzed using the Kolmogorov-Smirnov test, and the statistical differences between multiple groups were examined using one-way analysis of variance (ANOVA) with Bonferroni’s correction. A *p* value < 0.05 was considered to be statistically significant.

## Results

### KS23 ameliorated EAU progression

**Fig. 1:**
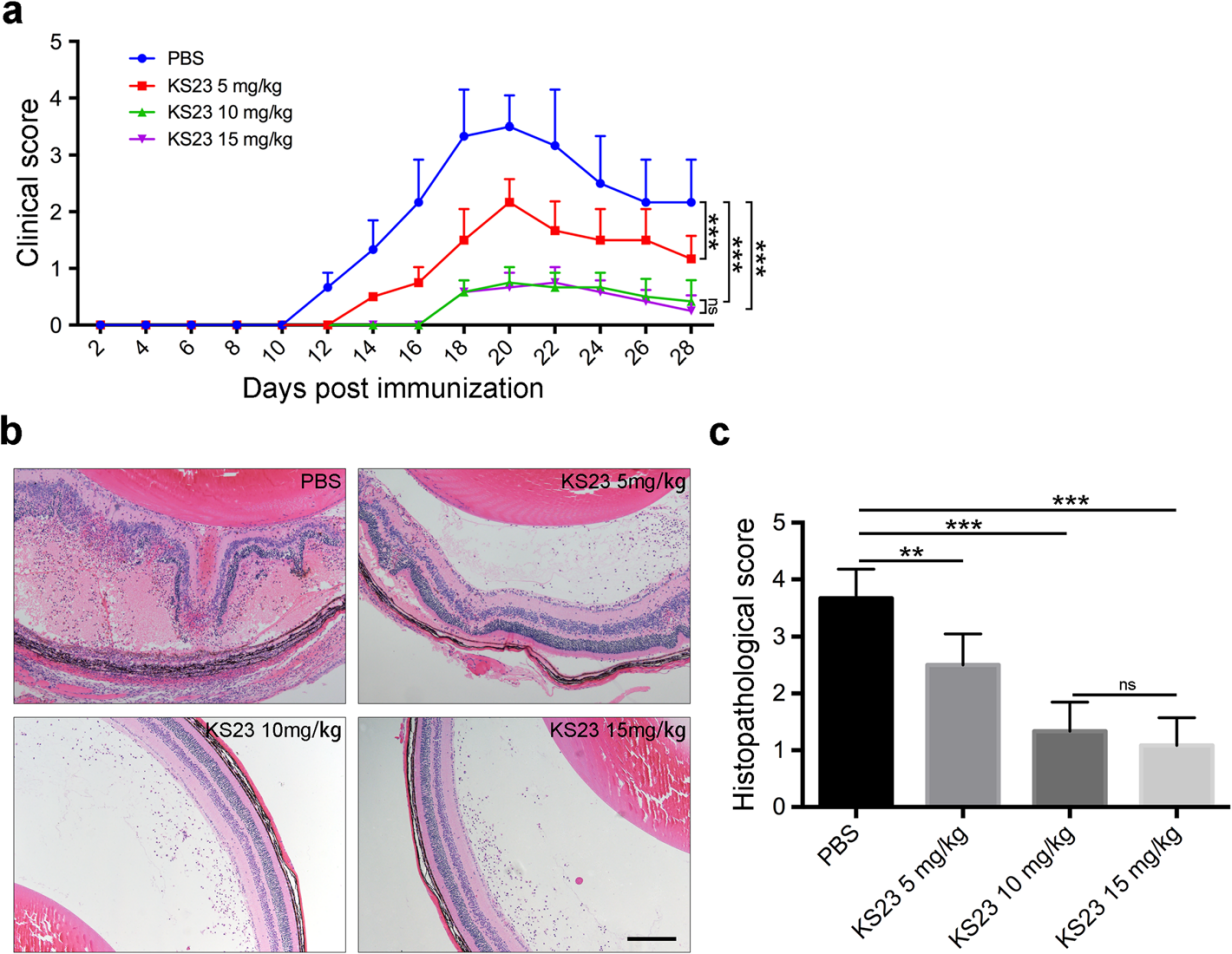
KS23 ameliorates the progression of EAU in a dose-dependent manner. B10RIII mice were induced with EAU using R161 and were sacrificed 28 days after immunization. KS23 (5 mg/kg, 10 mg/kg, or 15 mg/kg) was intraperitoneally injected every second day. *n* = 6 per group. **a** The mean clinical scores recorded every 2 days, from day 2 to day 28 after treatment with KS23. **b**, **c** Histological analysis of hematoxylin and eosin-stained retinal sections showing the extent of inflammation and the mean pathological scores. Scale bar 200 μm. Results are shown as means ± SD. **p < 0.01, ***p < 0.001; ns, non-significant

To study the effect that KS23 has on EAU, as well as the optimal dose of KS23, we intraperitoneally injected EAU mice with different concentrations of KS23. Our results show that compared to the EAU group, the onset of clinical symptoms was delayed by 2 days (5 mg/kg) or 6 days (10 mg/kg or 15 mg/kg) in the KS23-injected groups (Fig. 1a). We also determined that the peak clinical scores for all four experimental groups occurred 20–22 days after immunization. Meanwhile, clinical analysis revealed that the clinical scores for the three different KS23-injected groups were significantly lower than for the EAU group (5 mg/kg, *p* < 0.001; 10 mg/kg, *p* < 0.001; 15 mg/kg, *p* < 0.001) (Fig. 1a). Further, we determined that the histopathological scores for the EAU mice (3.67 ± 0.52) were significantly higher than those observed for the 5-mg/kg KS23-injected group (2.50 ± 0.55, *p* < 0.01), the 10-mg/kg KS23-injected group (1.33 ± 0.52, *p* < 0.001), and the 15-mg/kg KS23-injected group (1.08 ± 0.49, *p* < 0.001, Fig. 1b, c). However, the histopathological score for the 10-mg/kg KS23-injected group was not statistically different from that of the 15-mg/kg KS23-injected group. Based on these results, we chose a KS23 dose of 10 mg/kg to be administered every second day. We also chose day 21 after immunization as the observation time point, to decipher the mechanisms employed by KS23.

**Fig. 2:**
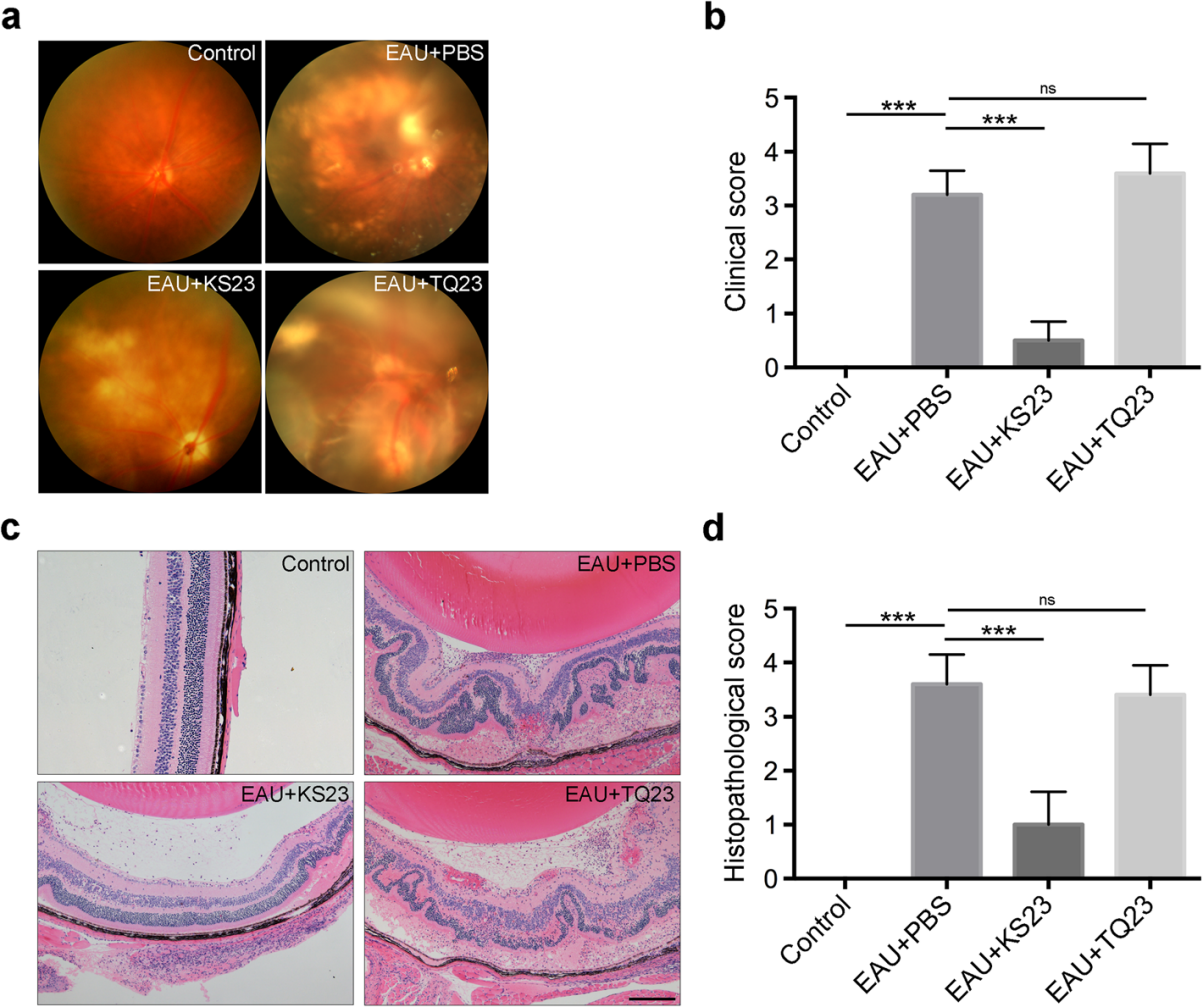
KS23 suppresses the development of EAU. Groups of B10RIII mice were immunized with R161 to induce EAU and treated with PBS, KS23, or TQ23. *n* = 5 per group. **a**, **b** Fundoscopy image and scores were obtained at day 21 after EAU was induced. **c**, **d** Histological examination and scores of eye sections performed on eyeballs collected on day 21. Scale bar 200 μm. Results are shown as means ± SD. ***p < 0.001; ns, non-significant

As seen in Fig. 2a, the fundus of EAU mice injected with PBS or TQ23 exhibited severe inflammation with focal or linear lesions, retinal vasculitis, vitritis, yellow-whitish retinal and choroidal infiltrates, retinal hemorrhage, and retinal detachment. In contrast, the fundus manifestations of the KS23-injected group were significantly improved, as compared to the EAU PBS- and TQ23-injected groups. The clinical score was also significantly increased in the PBS-injected EAU group (*p* < 0.001); however, it decreased following intraperitoneal injection with KS23 (*p* < 0.001, Fig. 2b). Histopathological studies of the retinas of the control group presented with near normal appearances, while those of the PBS-injected and TQ23-injected groups presented with numerous inflammatory cell infiltrates in the retina, as well as granulomas, photoreceptor layer damage, retinal foldings, retinal hemorrhage, and retinal detachment. Alternatively, the KS23-injected eyes maintained a relatively normal structure with only minimal cellular infiltrates observed (Fig. 2c). Likewise, the histopathological score for the KS23-injected group was significantly lower than that of the EAU group (*p* < 0.001) (Fig. 2d). Further, the histopathological score of the TQ23-injected group was not significantly different from the EAU group (*p* = 0.586) (Fig. 2d).

### KS23 reduced the expression of pro-inflammatory cytokines

**Fig. 3:**
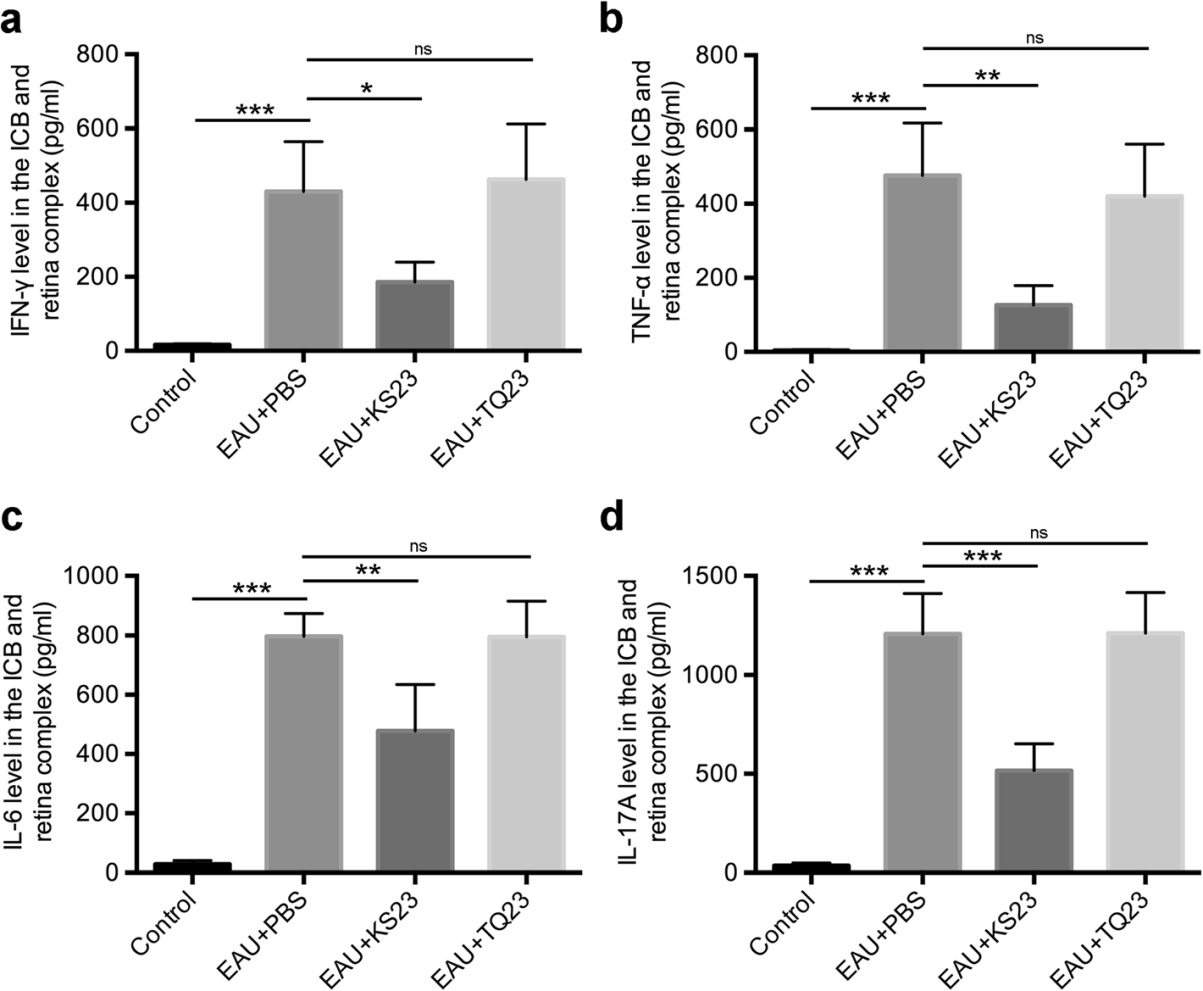
KS23 reduces the expression of pro-inflammatory cytokines. Groups of B10RIII mice were immunized with R161 to induce EAU and treated with PBS, KS23, or TQ23. *n* = 5 per group. IFN-γ (**a**), TNF-α (**b**), IL-6 (**c**), and IL-17A (**d**) expression levels in the ICB-retina complex, at day 21 after EAU was induced. Results are shown as means ± SD. *p < 0.05, **p < 0.01, ***p < 0.001; ns, non-significant

To assess the attenuating effect of KS23 on the immune response, expression levels of secreted IFN-γ, TNF-α, IL-6, and IL-17A in the ICB retinal complex were quantified. We determined that all of these inflammatory factors were significantly upregulated by EAU, as compared to the normal control group (*p* < 0.001, *p* < 0.001, *p* < 0.001, *p* < 0.001, respectively) (Fig. 3). Additionally, the expression of IFN-γ, TNF-α, IL-6, and IL-17A in the KS23-injected group was significantly lower than that in the EAU group (*p* < 0.05, *p* < 0.01, *p* < 0.01, *p* < 0.001, respectively). However, the mice intraperitoneally injected with TQ23 exhibited no significant difference in any of the examined inflammatory factors (Fig. 3).

### KS23 inhibited the expression of specific chemokines and chemokine receptors

**Fig. 4:**
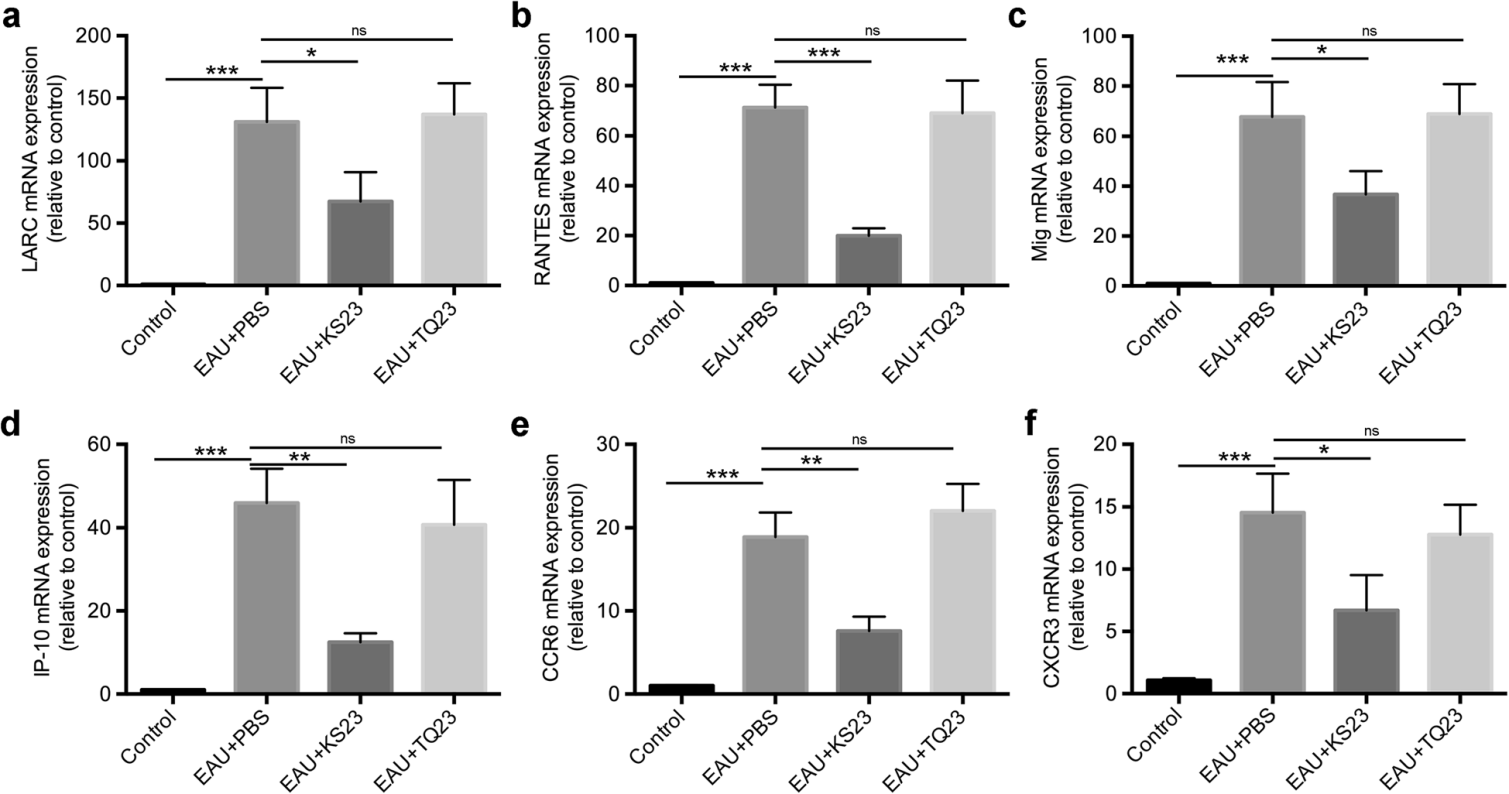
KS23 inhibited the expression of pro-inflammatory chemokines and chemokine receptors. Groups of B10RIII mice were immunized with R161 to induce EAU and treated with PBS, KS23, or TQ23. *n* = 5 per group. **a**–**f** The retinal expression level of LARC, RANTES, MIG, IP-10, CCR6, and CXCR3 were detected on day 21 following EAU induction, via qPCR. Results are shown as means ± SD. *p < 0.05, **p < 0.01, ***p < 0.001; ns, non-significant

To further evaluate the recruitment effect of KS23 for Th1 and Th17 cells into the retina, we analyzed the expression levels of specific chemokines and chemokine receptors by qRT-PCR. The mRNA expression of LARC, RANTES, MIG, IP-10, CCR6, and CXCR3 were all found to be significantly upregulated in the EAU group, as compared to that of the control group (*p* < 0.001, respectively) (Fig. 4). These results were consistent with the decreased level of retinal inflammation observed in EAU mice injected with KS23, in contrast to those injected with PBS. Further, the expression of every detected chemokine and chemokine receptor was markedly downregulated in the KS23-injected group (*p* < 0.05, *p* < 0.001, *p* < 0.05, *p* < 0.01, *p* < 0.01, *p* < 0.05) (Fig. 4). Alternatively, EAU mice intraperitoneally injected with TQ23 exhibited no significant suppression of the chemokines or chemokine receptors.

### KS23 inhibits the proportions of peripheral Th1 and Th17 subsets

**Fig. 5:**
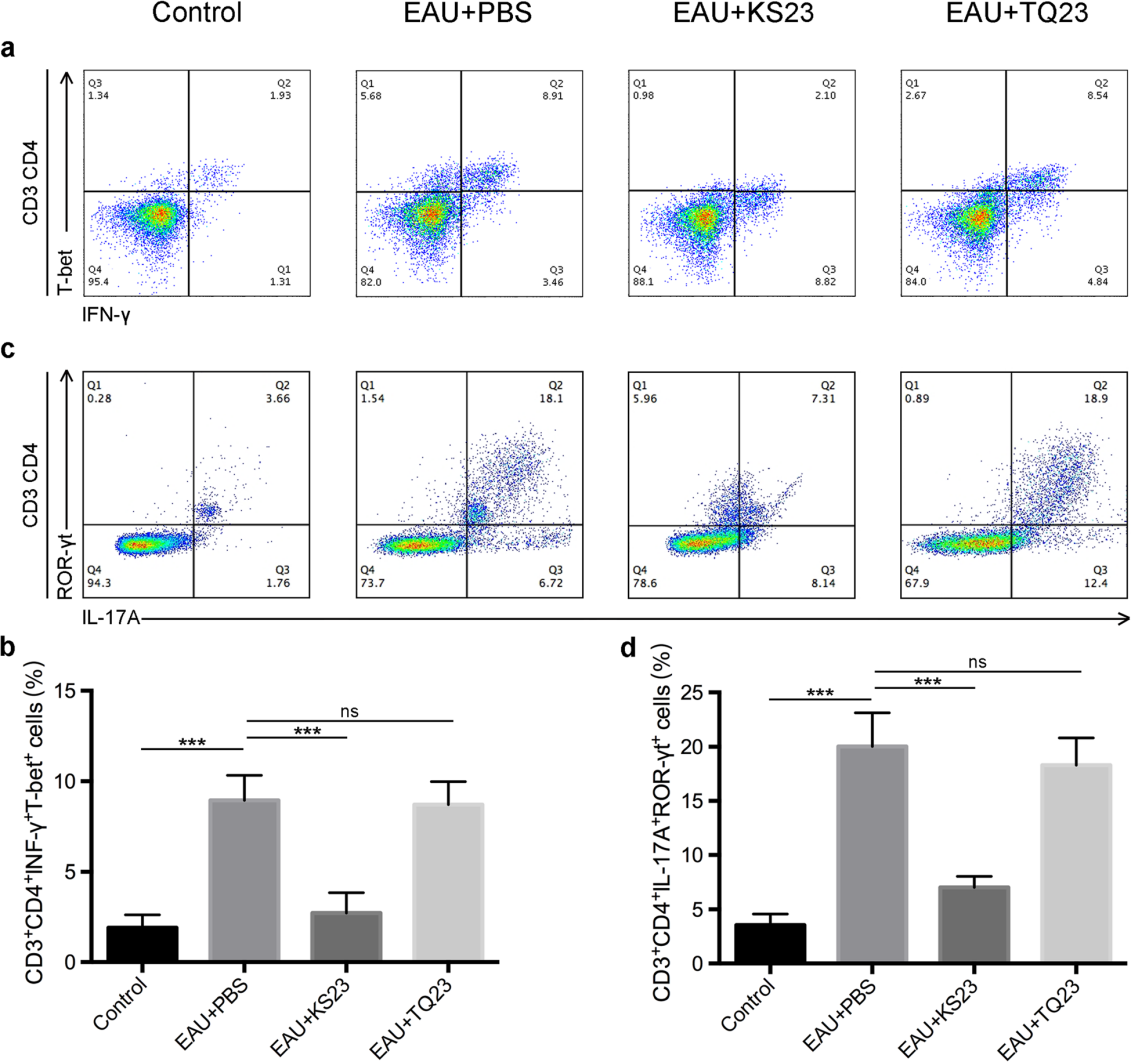
KS23 inhibits the proportions of peripheral Th1 and Th17 subsets. Groups of B10RIII mice were immunized with R161 to induce EAU and treated with PBS, KS23, or TQ23. *n* = 6 per group. Flow cytometric analysis of Th1(**a**) (IFN-γ^+^T-bet^+^) and Th17 (**c**) cells (IL-17A^+^RORγt^+^) in CD3^+^CD4^+^ T cells, isolated from the spleens of mice on day 21 after EAU induction. The proportion of Th1 (**b**) and Th17 (**d**) cells in the splenic cells of mice on day 21 after EAU induction. Results are shown as means ± SD. *p < 0.05, **p < 0.01, ***p < 0.001; ns, non-significant

To investigate the mechanism employed by KS23 to modulate the inflammatory immune response, lymphocytes from the mouse spleens were collected. Results show that the Th1 and Th17 cells of EAU mice spleens were higher in proportion, than those observed in the control group (*p* < 0.001, *p* < 0.001) (Fig. 5). Moreover, the proportion of Th1 and Th17 cells in TQ23-injected mice was similar to that of the EAU group (Fig. 5). Alternatively, compared with the EAU group, KS23-injected mice had fewer Th1 cells (*p* < 0.001) and Th17 cells (*p* < 0.001) present in their spleens (Fig. 5). No significant differences were found in the proportion of Treg cells among the EAU group, KS23-injected group, and TQ23-injected group (Additional file 2: Figure S2).

### KS23 activates AMPK and suppresses the NF-κB signaling pathway

**Fig. 6:**
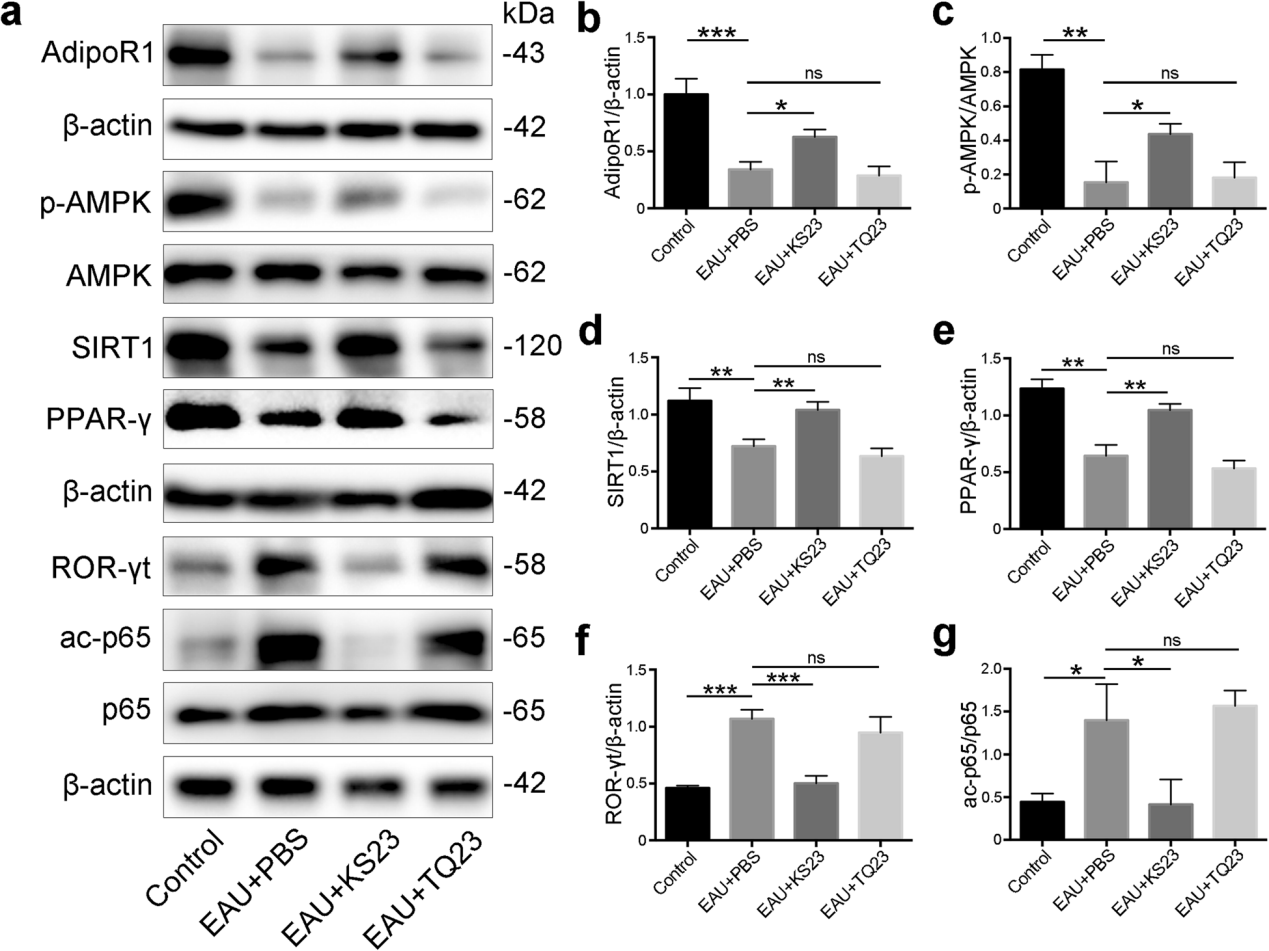
KS23 activates AMPK and suppresses the NF-κB signaling pathway. Groups of B10RIII mice were immunized with R161 to induce EAU and treated with PBS, KS23, or TQ23. *n* = 5 per group. **a** Western blot analysis of the expression of AdipoR1, phospho-AMPK, AMPK, SIRT1, PPARγ, RORγt, acetylated p65, and p65 in the retina of EAU mice. The band intensities were assessed using ImageJ. **b** AdipoR1 expression levels are represented as their ratios compared to β-actin. **c** phospho-AMPK levels are represented as the ratio of phospho-AMPK to AMPK. **d**–**f** SIRT1, PPARγ, and RORγt expression levels are represented as their ratios compared to β-actin. **g**. The acetylation level of p65 is represented as the ratio of acetylated p65 to p65. Results are shown as means ± SD. *p < 0.05, **p < 0.01, ***p < 0.001; ns, non-significant

APN has been shown to activate the AMPK signaling pathway and limit autoimmune inflammation, via induction of SIRT1, PPARγ, and RORγt expression [16, 22]. To determine whether the mechanism employed by KS23 to ameliorate EAU is similar to that of APN, we quantified the expression of AdipoR1, phospho-AMPK, AMPK, SIRT1, PPARγ, and RORγt in the retina using western blot analysis. As the results show in Fig. 6a, the EAU group expressed significantly lower levels of AdipoR1, phospho-AMPK, SIRT1, and PPARγ, compared to those of the control group (*p* < 0.001, Fig. 6b; *p* < 0.01; Fig. 6c–e, respectively). The suppression of these four proteins resulted in subsequent upregulation of ROR-γt (*p* < 0.001; Fig. 6f), which is the key transcription factor for Th17 differentiation [23]. However, intraperitoneally injecting the EAU mice with KS23 resulted in increased expression of AdipoR1 (*p* < 0.05; Fig. 6b). AMPK was found to be upregulated via phosphorylation (*p* < 0.05; Fig. 6c), and the expression of SIRT1 and PPARγ was upregulated as well (*p* < 0.01; Fig. 6d, e, respectively), thereby suppressing RORγt expression (*p* < 0.001; Fig. 6f). Alternatively, the increased expression of SIRT1 resulted in the deacetylation of p65 (*p* < 0.05; Fig. 6g).

## Discussion

The traditional treatments for autoimmune uveitis are corticosteroids and systemic immunosuppressive agents, which generally cause adverse side effects [24]. To avoid these negative effects and to increase therapeutic effectiveness, development of therapies that target the molecular pathways responsible for induction of uveitis pathogenesis is desirable. Similar to other published studies, our results support the notion that the autoimmune responses in the eye, as well as in other tissues, are mediated by both the Th1 and Th17 effectors. Numerous studies have described a key role of IL-17 in the development of autoimmune diseases. Moreover, many clinical trials have been completed that determined the efficacy of anti-IL-17 agents as therapeutic options for these conditions. However, the therapeutic effect was found to be minimal for autoimmune diseases [13]. The inherent plasticity of immune responses may have been one factor that contributed to these poor results. Furthermore, Th1 and Th17 cells derived from the same Ag-specific precursors can differentiate along either pathway, and thus, therapeutic inhibition of one of these Th cell subsets may induce one effector pathway to take over the other [25]. For example, deficiency or neutralization of IFN-γ, the primary regulatory cytokine for Th1, results in the elevation of Th17, and vice versa [9, 26, 27]. Hence, the most effective treatment should not only target one of these cell subsets, but rather, both simultaneously.

Although a previous study demonstrated that APN activates both Th1 and Th17 cells under physiological conditions [28], other reports have shown that APN negatively affects the activation of T lymphocytes in autoimmune diseases [29–31]. Moreover, a previous study has claimed that recombinant APN can prevent abortion by modulating the Th17/Treg imbalance through the p38 mitogen-activated protein kinase (MAPK)-signal transducer and activator of transcription 5 (STAT5) pathway [32].

Unfortunately, the adverse side effects associated with overexpression of APN included decreased bone density, weight gain, and infertility [33]. The transgenic mouse with increased serum full-length adiponectin showed a moderate increase in body weight and infertility [34]. Also, these transgenic mice had inferior mechanical properties and lower femur bone mineral content than the control mice [35]. However, the gAPN induced dramatic and sustainable weight reduction of mice consuming a high-fat/sucrose diet [18]. Meanwhile, bone mineral density measurements, bone histomorphometry, and histology of mice overexpressing gAPN showed no differences to the controls [36]. Consequently, we inferred that full-length adiponectin was more likely to cause side effects than gAPN. Hence, the development of an agent that maintains the beneficial effects of gAPN without inducing the detrimental side effects is an important task. Using peptides in these therapeutic formulations is preferable to using proteins, because peptides exhibit higher activity per unit mass, lower immunogenicity, better organ penetration, higher solubility in water, greater stability during storage, and more stable production methods [37]. Furthermore, peptide-based immunotherapy has been successfully described in various experimental animal models [38, 39]. In the current study, KS23 was employed as a novel 23-amino-acid peptide derived from the globular C-terminal domain of APN. In our EAU mouse model, KS23 was shown to substantially suppress the clinical and histopathological scores, as well as the expression of pro-inflammatory cytokines. To determine the primary mechanism used by KS23 in the amelioration of uveitis pathogenesis, we focused on the Th cells, which were recognized as a primary contributor to the etiology of autoimmune uveitis.

In our study, we discovered that intraperitoneal injection with KS23 reduced the proportion and number of Th1 and Th17 cells in the spleens of EAU mice. We also determined that KS23 caused an increase in the expression of AdipoR1 as well as AMPK phosphorylation, which subsequently upregulated the expression of SIRT1 and PPARγ, thereby reducing the expression of RORγt. AMPK plays a crucial role in the regulation of cellular energy metabolism and SIRT1-dependent regulation of the anti-inflammatory response [40]. Furthermore, previous reports have demonstrated that PPARγ selectively reduces Th17 differentiation through suppressing RORγt expression in CD4^+^ T cells [23]. AMPK, the upstream regulator of SIRT1 and PPARγ, induces the expression of PPARγ through its phosphorylation [41]. In addition, there are three type of receptors for APN, namely AdipoR1, adiponectin receptor 2 (AdipoR2), and T-cadherin [42]. AdipoR1 is expressed in the retinas of both humans and mice, and when APN binds to AdipoR1, the AMPK pathway becomes activated [22, 43]. Also, since RORγt is the key transcription factor for Th17, KS23 may have functioned to downregulate the ratio and number of Th17 cells via inhibition of RORγt expression through regulation of the AMPK pathway. These results were in accordance with those reported by Zhang et al., which indicated that APN significantly suppressed Th 17 cell differentiation, while also acting to decrease the overall inflammatory response in an experimental model of autoimmune encephalomyelitis, via regulation of the SIRT1/PPARγ/RORγt pathway [16]. Although there was a study which reported that recombinant adiponectin ameliorated abortion in mice by regulating Th17/Treg [32], however, in our study, it was demonstrated that KS23 could not affect the frequency of Treg cells in the lymphocytes from spleens. We inferred that the full-length APN but not KS23 which is derived from globular C-terminal domain of APN can regulate Th17/Treg.

Since NF-κB is one of the most important stimulators of the production and activation of pro-inflammatory cytokines, we also sought to examine the effect that KS23 had on NF-κB in EAU models. Previous studies have demonstrated that SIRT1 acts to directly deacetylate the RELA/p65 subunit of the NF-κB complex, thereby inactivating the transcription of NF-κB-dependent gene and suppressing pro-inflammatory immune responses [44–46]. According to our results, KS23 may also act to ameliorate autoimmune uveitis via an anti-inflammatory response induced by the deacetylation of RELA/p65, following the upregulation of AMPK and SIRT1 expression.

Uveitis is characterized by aberrant infiltration of leukocytes into the uvea, retina, vitreous humor, and sclera. To study the molecular mechanisms that may cause a decrease in the specific recruitment of Th1 and Th17 cells into the retina of KS23-injected mice during EAU, we examined the expression levels of specific chemokines (LARC, MIG, and IP-10) as well as that of the Th1- and Th17-specific chemokine receptors (CXCR3 and CCR6) in retinal tissue. RANTES, specifically, is a chemokine that recruits basophils, eosinophils, and T cells to the sites of inflammation [47]. These chemokines and chemokine receptors were all found to be upregulated in the retinas of EAU mice, as compared to the normal control mice. However, they were all negatively regulated by KS23 in EAU mice. In addition, on further examination of the cytokine profiles, a reduction in the expression of IFN-γ, TNF-α, IL-6, and IL-17A was observed in ICB and retinal samples following injection with KS23 in EAU mice, when compared to EAU mice injected with PBS.

## Conclusions

The marked anti-inflammatory capacity of KS23 as compared to TQ23 was shown in EAU. Furthermore, EAU mice that were injected with KS23 exhibited milder characteristic manifestations of uveitis. These beneficial effects were the result of reduced pro-inflammatory cytokines, chemokines, and chemokine receptors in conjunction with the suppression of Th1 and Th17 cell proportion, through activation of the AMPK pathway and simultaneous inhibition of the NF-κB signaling pathway. Taken together, these results suggest that KS23 may serve as a promising novel prevention agent for autoimmune uveitis. The direct molecular target of KS23 remains unclear, as does whether KS23 is able to interact directly with AdipoR1. Nevertheless, successful prevention of EAU with KS23 serves to aid the discovery and development of a new potential therapy for uveitis patients.

## Supplementary information

**Additional file 1: Figure S1.** Gating strategy to identify CD3^+^CD4^+^ lymphocyte. Forward and side scatter (FSC and SSC) gating on area (A) and height (H) gated lymphocyte and excluded debris and non-single cell events. Then FSV510^−^ cells were gated as live cells and subsequently CD3^+^CD4^+^ lymphocytes were gated.

**Additional file 2: Figure S2.** KS23 performs no significant effect on the proportions of peripheral Treg subsets. Groups of B10RIII mice were immunized with R161 to induce EAU and treated with PBS, KS23 or TQ23. *n* = 6 per group. a. Flow cytometric analysis Treg (CD25^+^Foxp3^+^) in CD3^+^CD4^+^ T cells isolated from the spleens of mice on day 21 after EAU induction. b. The proportion of Treg cells in the splenic cells of mice on day 21 after EAU induction. Results are shown as means ± SD. *p < 0.05; ns, nonsignificant.

## Data Availability

The datasets analyzed during the current study are available from the corresponding author on reasonable request.

## Abbreviations

Ac-NF-κB: Acetyl-NF-κB
AdipoR1: Adiponectin receptor 1
AdipoR2: Adiponectin receptor 2
AMPK: AMP-activated protein kinase
ANOVA: Analysis of variance
APN: Adiponectin
BRB: Blood-retinal barrier
BSA: Bovine serum albumin
CCR6: C-C motif chemokine receptor 6
cDNA: Complementary DNA
CXCR3: C-X-C motif receptor 3
EAU: Experimental autoimmune uveitis
ECL: Enhanced chemiluminescence
ELISA: Enzyme-linked immunosorbent assay
gAPN: Globular APN
H&E: Hematoxylin and eosin
ICB: Iris ciliary body
IFN-γ: Interferon gamma
IL-17A: Interleukin-17A
IL-6: Interleukin-6
IP-10/CXCL10: IFN-γ-inducible protein 10
IRBP161–180 (R161): Interphotoreceptor retinoid binding protein 161–180
LARC/CCL20: Liver and activation-regulated chemokine
MAPK: Mitogen-activated protein kinase
MIG/CXCL9: Monokine induced by gamma interferon
NF-κB: Nuclear factor-kappa B
PPAR-γ: Peroxisome proliferator-activated receptor gamma
PVDF: Polyvinylidene difluoride
qRT-PCR: Quantitative real-time polymerase chain reaction
RANTES/CCL5: Regulated upon activation, normal T cell expressed, and secreted
ROR-γt: RAR-related orphan receptor gamma t
SD: Standard deviation
SDS-PAGE: Sodium dodecyl sulfate-polyacrylamide gel electrophoresis
SIRT1: Sirtuin 1
STAT5: Signal transducer and activator of transcription 5
Th: T helper cells
Th1: Th type 1 cells
Th17: Th type 17 cells
TNF-α: Tumor necrosis factor alpha
Treg: T regulatory cells
