# Supplementary figures and images for: KS23, a novel peptide derived from adiponectin, inhibits retinal inflammation and downregulates the proportions of Th1 and Th17 cells during experimental autoimmune uveitis
Source: J Neuroinflammation. 2019 Dec 28;16:278. doi: 10.1186/s12974-019-1686-y (PMC6935244; doi:10.1186/s12974-019-1686-y)

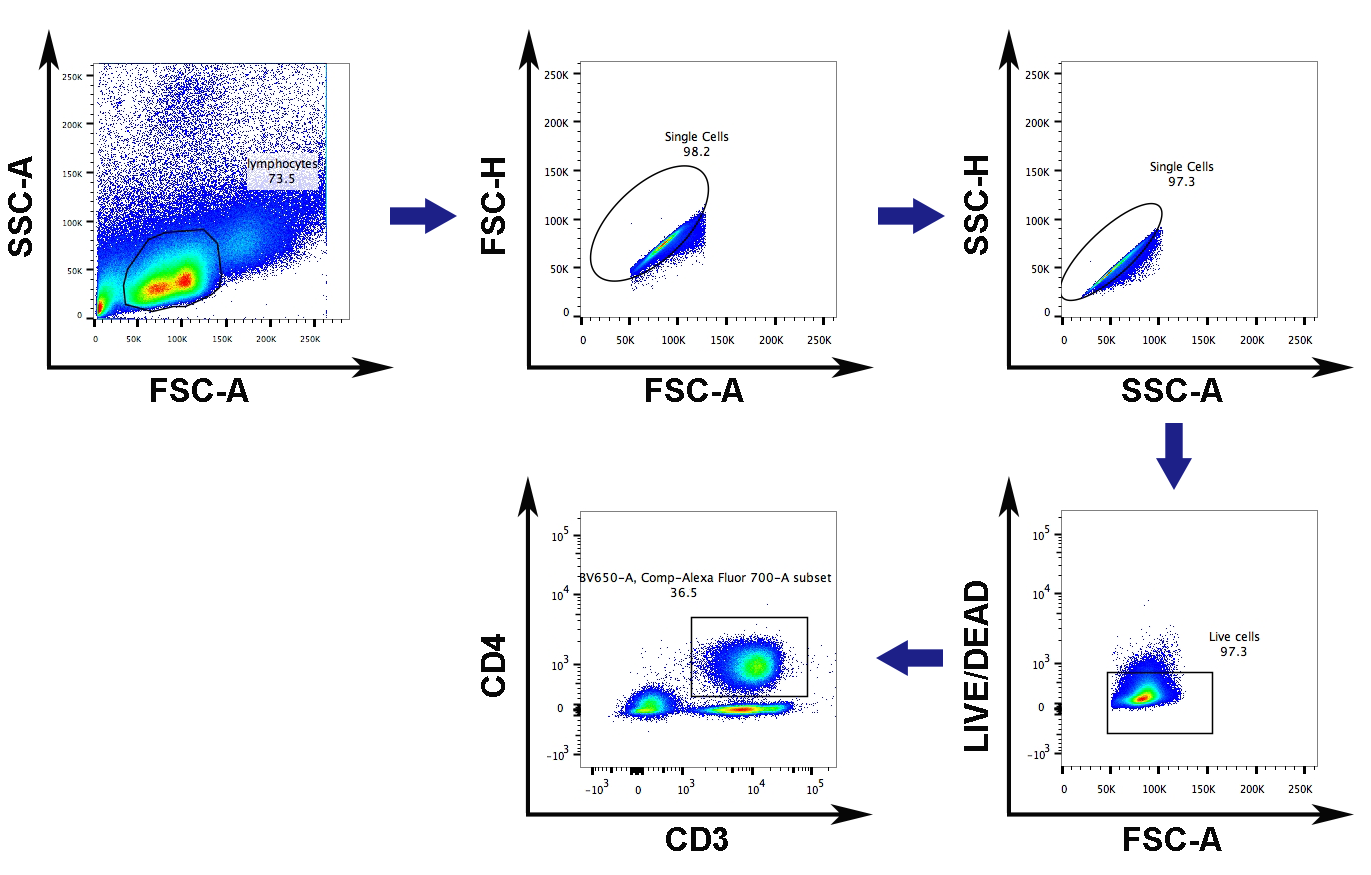

Supplement: Supplementary file 1 — Additional file 1: Figure S1. Gating strategy to identify CD3+CD4+ lymphocyte. Forward and side scatter (FSC and SSC) gating on area (A) and height (H) gated lymphocyte and excluded debris and non-single cell events. Then FSV510− cells were gated as live cells and subsequently CD3+CD4+ lymphocytes were gated. [file 12974_2019_1686_MOESM1_ESM.tif]

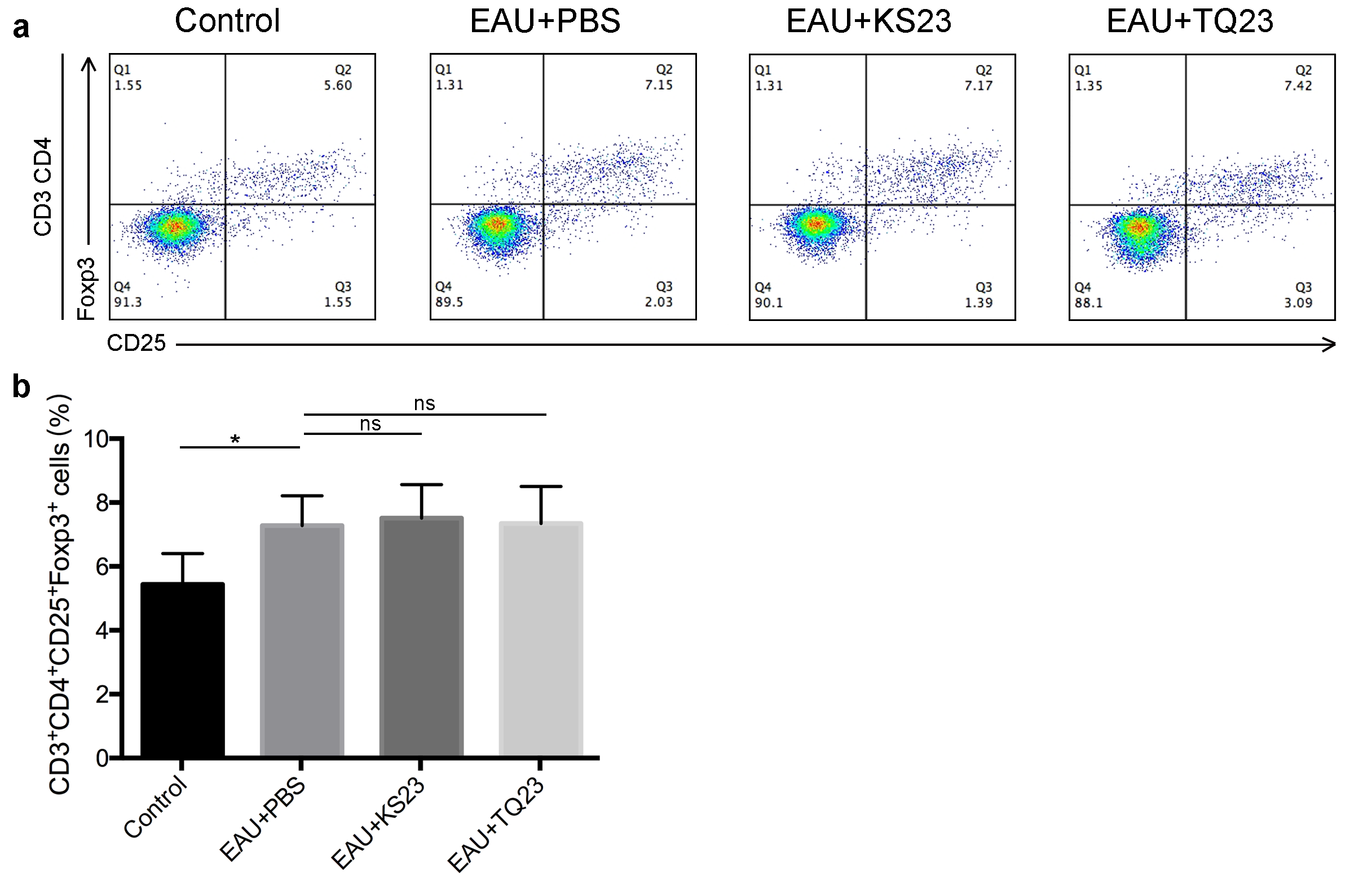

Supplement: Supplementary file 2 — Additional file 2: Figure S2. KS23 performs no significant effect on the proportions of peripheral Treg subsets. Groups of B10RIII mice were immunized with R161 to induce EAU and treated with PBS, KS23 or TQ23. n = 6 per group. a. Flow cytometric analysis Treg (CD25+Foxp3+) in CD3+CD4+ T cells isolated from the spleens of mice on day 21 after EAU induction. b. The proportion of Treg cells in the splenic cells of mice on day 21 after EAU induction. Results are shown as means ± SD. *p < 0.05; ns, nonsignificant. [file 12974_2019_1686_MOESM2_ESM.tif]
